# Social media as a platform for health-related public debates and discussions: the Polio vaccine on Facebook

**DOI:** 10.1186/s13584-016-0093-4

**Published:** 2016-11-10

**Authors:** Daniela Orr, Ayelet Baram-Tsabari, Keren Landsman

**Affiliations:** 1 Faculty of Education in Science and Technology, Technion – Israel Institute of Technology, Haifa, Israel; 2 Department of Community Medicine and Epidemiology, Carmel Medical Center, Haifa, Israel

**Keywords:** Social media, Facebook, Vaccination debates and discussions, Polio

## Abstract

**Background:**

Social media can act as an important platform for debating, discussing, and disseminating information about vaccines. Our objectives were to map and describe the roles played by web-based mainstream media and social media as platforms for vaccination-related public debates and discussions during the Polio crisis in Israel in 2013: where and how did the public debate and discuss the issue, and how can these debates and discussions be characterized?

**Method:**

Polio-related coverage was collected from May 28 to October 31, 2013, from seven online Hebrew media platforms and the Facebook groups discussing the Polio vaccination were mapped and described. In addition, 2,289 items from the Facebook group “Parents talk about Polio vaccination” were analyzed for socio-demographic and thematic characteristics.

**Results:**

The traditional media mainly echoed formal voices from the Ministry of Health. The comments on the Facebook vaccination opposition groups could be divided into four groups: comments with individualistic perceptions, comments that expressed concerns about the safety of the OPV, comments that expressed distrust in the Ministry of Health, and comments denying Polio as a disease.

In the Facebook group “Parents talk about the Polio vaccination”, an active group with various participants, 321 commentators submitted 2289 comments, with 64 % of the comments written by women. Most (92 %) people involved were parents. The comments were both personal (referring to specific situations) and general in nature (referring to symptoms or wide implications). A few (13 %) of the commentators were physicians (*n* = 44), who were responsible for 909 (40 %) of the items in the sample. Half the doctors and 6 % of the non-doctors wrote over 10 items each. This Facebook group formed a unique platform where unmediated debates and discussions between the public and medical experts took place.

**Conclusion:**

The comments on the social media, as well as the socio-demographic profiles of the commentators, suggest that social media is an active and versatile debate and discussion-facilitating platform in the context of vaccinations. This paper presents public voices, which should be seen as authentic (i.e. unmediated by the media or other political actors) and useful for policy making purposes. The policy implications include identifying social media as a main channel of communication during health crises, and acknowledging the voices heard on social media as authentic and useful for policy making. Human and financial resources need to be devolved specifically to social media. Health officials and experts need to be accessible on social media, and be equipped to readily provide the information, support and advice the public is looking for.

## Background

In March 2013, a type 1 wild Polio virus (WPV1) was found during routine environmental surveillance of the sewage system in a southern town in Israel^1^ [32]. Epidemiological analysis showed that children under 9 years of age were the carriers and the main distributers of the virus [44].

Children in Israel are regularly vaccinated with Inactivated Poliovirus Vaccine (IPV) which protects them from developing Poliomyelitis but does not prevent them from becoming transmitters and spreading the disease if infected with the virus, nor from spreading the virus [43]. Older citizens of Israel received both the IPV and the live (attenuated) Oral Polio Vaccine (OPV), which were routinely given to all infants between 1990^2^ and 2005 [44]. The WHO declared Israel Polio-free on June 21, 2002 [44]. The OPV was removed from routine vaccination and replaced by IPV, due to its better safety profile.^3^

Following the isolation of WPV1 from sewage samples in March 2013, on August 5, 2013 the Ministry of Health launched a vaccination campaign called “Two drops” targeted at children in southern Israel [24]. However, two weeks later, when analysis showed that the Polio virus had circulated more widely, the campaign was extended to the entire country [20]. OPV was only administered to children who had already been vaccinated with IPV. This additional vaccination was in fact meant to protect society more than the children themselves. In other words, OPV was added to prevent children from becoming carriers of WPV1 and from spreading the virus, or to promote herd immunity. Even though the scientific consensus which led to the recommendation to add OPV to young children’s vaccination routine was very solid [24, 26], this guideline sparked intense public debates and discussions, which were widely reviewed in the traditional as well as electronic mass media and in the social media^4^ including platforms such as Facebook, various online forums, and blogs.

By November 2013, Israel’s sewage samples all came back negative for wild Polio virus [26]. By January 2014, Israel’s official national campaign was over [20]. Overall, 945,000 children constituting 78.75 % of the target population were vaccinated with the OPV during the 2013 campaign [23]. By the end of the campaign, the Israeli Ministry of Health announced that the OPV would once again become the routine vaccination for children in Israel.^5^ Since the campaign was initiated, and to this day, there has not been a single case of Polio virus [24]. In April 2015, Israel was officially once again Polio-free, as declared by the World Health Organization.^6^

Scientific information is playing a growing role in the social media [30]. Numerous debate and discussion groups have emerged to address scientific topics [1, 13]. The reliance on social media is one of the newer uses of online resources more generally; as of 2014 the Internet surpassed television as Americans’ primary source of information about science and technology. Social media are also a rapidly expanding feature of online life: today, 65 % of American adults use social media [40]. Facebook is the most popular and frequently used social media platform among American 13 to 17 year olds, with 71 % of all teens using the platform [29]. Facebook had one billion daily active users on average in September 2015.^7^ Of these users, 700 million are members of Facebook groups [34].

As a result of this emergent association between science communication and social media, scientific magazines such as ‘Science’ and ‘PNAS’ have recently issued calls to better understand how online environments affect the communication of science information to the public. A number of researchers have drawn attention to the paucity of systematic empirical explorations of the ways in which the new media in general and social media in particular have changed the science communication landscape [6, 7]. It has been shown, for example, that people use Facebook as an information source for socio-scientific issues [16]. But how do people integrate online science resources into their decision- making and meaning- making processes?

In general, public participation in scientific issues, and public understanding of scientific information are essential to modern society. Public engagement with health-related information is even more so. In 2013, 59 % of all American adults searched for health information online and of these, 16 % looked for others who shared the same health concerns [14]. In France, almost half of all Web users aged 15–30 used the Internet for health purposes [3]. A recent paper exploring health information seeking in Europe showed that social media can act as a complementary source of information to traditional and online media [25]. Individuals who showed an inclination to use social media in conjunction with other channels considered it more important to be well informed, were more motivated to find additional information, and were more sensitive to risks in general [25]. In Israel, there are several online forums and social media outlets which support patients and people interested in health issues. One for example, is “Kamoni”, an online health forum where patients can find others with similar health problems and seek advice and consultation from experts. This forum is mainly oriented toward patients with chronic illnesses. In this paper, we show that in non-chronic situations as well, social media can act as a health information seeking and providing resource, in addition to a space for non-mediated debate, discussion, and support.

Internationally, social media have been found to play a profound role in health promotion [9, 37], where, unlike in Israel, other social media outlets, such as Twitter, are equally popular. For example, the Facebook and Twitter accounts of the Center for Disease Control (CDC) are comparable in terms of number of users (559,987 users for CDC official Facebook page vs. 645,121 followers for the CDC gov. Twitter account respectively).

In 2012, the journal ‘Vaccine’ devoted a special issue to the role of internet use in making vaccination decisions.^8^ The articles in that issue all stressed the potential of the social media to serve as an essential agent in influencing and shaping scientific and medical decisions [4].

In the Israeli context, the Israeli media 2013 report [31] indicated that the social media platform Facebook is the most widely used social media site in Israel, and spans users with diverse socio-economic backgrounds. In Israel, there are about 4 million Facebook users, 2.4 of whom check their Facebook account on a daily basis. Of these, 52 % are females. Many users are in the 18–24 age range (28 %), followed by the 25–34 (27 %), 35–44 (15 %), 13–17 (13 %), 45–54 (9 %), and 55 and over (about 9 %) age brackets [31].

Only a few scholarly papers have provided a thorough description of the contemporary social media landscape in Israel in the context of health and science communication [15, 20, 38]. However, documentation shows that 56 % of all online users in Israel read about a medical issue online before going to the doctor [31]. This article analyzes the role played by social media as a public platform in vaccination-related debates and discussions. We present data collected from the Israeli mainstream media and Facebook focusing on a specific content-driven group called “Parents talk about Polio vaccination”, the only diversified Facebook Polio-related group. The exploration of the texts submitted and shared by this particular group provides a unique opportunity to analyze authentic exchanges while the debates and discussions were ongoing. From this point on, when we use the term *authentic*, we mean that an authentic public voice is unmediated by the media or other political actors.

## Objective

Our objectives were to map and describe the role played by social media and mainstream web-based media as platforms for vaccination-related public debates and discussions during the Polio crisis in Israel in 2013.

## Method

### Primary mapping of the research field

To map the research field, we manually collected the Polio-related coverage in ten online Hebrew platforms between May 28 and October 31, 2013. During this period the Polio crisis was at its height. According to a “Google trends” search, searches for the term “Polio” (in Hebrew), peaked from the end of May until November 2013.^9^ The choice of general media platforms (not their social media pages) was made on the basis of popularity and diversity of audiences. According to TGI data relevant to 2013, online general media platforms were used on a daily basis by 5.2 million Israeli users [31]. Of the ten platforms we analyzed, seven were mainstream popular media news sites: Ha’aretz, Israel Hayom, NRG, Mako, The Marker, Walla, and Ynet; one was a popular science blog called Sof Ha-Olam-Mabat me-ha-yaziah (“End of the world: a view from the balcony”), one was an open Facebook page called Vaccinations Inc., and one was the most popular news site serving the ultra-orthodox sector in Israel called Be-hadrei Haredim. The items were chosen according to the search term “Polio”, and were identified manually as discussing the government drive to administer children the OVP Polio vaccination as the main theme. Since a search for online and social media discussing the Polio vaccine crisis in Arabic did not yield sufficient resources, only Hebrew-language platforms were examined for the study.

This exploration resulted in 235 items overall: Ynet (56 items), Mako (35 items), NRG (30 items), Israel Hayom (30 items), Walla (29 items), Ha’aretz (27 items), Be-hadrei Haredim (11 items), Vaccinations Inc. (10 posts), The Marker (4 items), Sof Ha-Olam-Mabat me-ha-yaziah (3 posts). The items were then grouped by their main subject or headline.

### Social media mapping

Social media outlets such as Twitter and YouTube are not as popular in Israel as Facebook [31]. Therefore, we chose Facebook as the social media outlet from which to collect data. We mapped Facebook for groups discussing the Polio vaccination and found five main groups with a flourishing discussion: “Parents talk about the Polio vaccination”, “Mamazone” (89,977 members), a general group dedicated to mothers, “Mothers Say No to the Attenuated Polio Vaccination” (4626 members), “Open Notepad – Parents for Transparency and Safety in Vaccinations” (4798 members) and “Vaccines Inc.” (6200 members). We elaborate on the three latter groups in the results section. Mamazone, a closed group, could not be accessed. All member counts are relevant to October 21, 2015. Out of all the Facebook groups discussing the Polio vaccinations, we chose for further analysis the group “Parents talk about the Polio vaccination”, which proclaimed itself neutral, and had the most diverse range of participants to analyze.

### Data collection from the Facebook group “Parents talk about the Polio vaccination”

Data were collected from the “Parents talk about the Polio vaccination” group from August 14 to November 12, 2013. Although the group continued to be active after November 12 (December 2013-February 2014), this period of time covered the most intensive activity, with 1,039 posts and their respective comments.

When collecting the data on this group, an ‘item’ referred to a single post or a single comment. The sampling frame was created using a data-mining technique; a PHP script available from the Facebook site. This script served to collect each post’s first 25 comments.^10^ This can be viewed as a limitation, since not all comments for every post were analyzed. It is, however, not a strong limitation since each post or comment was analyzed as an individual unit. From the sampling frame, a sample of 2,289 items were randomly selected using a ‘randomize numbers’ command. This was a representative sample of the initial sampling frame.

All the data were anonymized and are available for scientific use. This methodology may give rise to ethical concerns, given that the products of human behavior are scrutinized. Nevertheless, according to the Codes of Ethics and Conduct of Internet Research [2], if an observation of public behavior takes place in public situations where subjects would expect to be observed by strangers (such as an open ‘Facebook’ discussion), explicit individual consent is not required. IRB approval from the authors’ affiliate institutes’ Ethics Committees were obtained nonetheless. We based our conclusions solely on data collected from open group discussions and open profiles.

The first two authors conducted topical and statistical analyses of the socio-demographic variables. The third author was one of the founders of the group “Parents talk about the Polio vaccine”, though not one of the administrators of the group. Today she is an active member of a Facebook group called “Talking about vaccines”. The third author was not involved in the data gathering and data analysis processes; hence bias should not be assumed.

This paper has a descriptive aim. All social media materials – in other words the various Facebook groups – were thematically analyzed [46] by the first author for emerging themes. Themes were not identified in advance but rather derived from the data [28]. This qualitative technique of emerging themes is prominent in the social sciences, and leans on the ethnographic assumption that richness of data can be achieved by “letting the data speak for itself” [8, 11]. The data were analyzed manually. The unit of analysis consisted of a single comment or post in a Facebook discussion. The coding was done inductively,such that general codes were defined by aggregating specific trends [41]. Inter-coder reliability on nearly 10 % of the sample of 2,289 items (200 items) collected from the group “Parents talk about Polio vaccination”, was satisfactory with a Cronbach’s alpha of 0.75. The codes selected for the reliability process were all codes used for the coding of items from that group. A trained coder and the first author coded 200 items, and then compared the results between them, and ran a Cronbach’s alpha test.

## Results

Two hundred and thirty-five Polio-related news items collected from seven mainstream popular media news sites (Ha’aretz, Israel Hayom, Mako, NRG, The Marker, Walla, and Ynet) primarily contained information sourced from the Ministry of Health (87 %), including interviews with officials and guidelines (for example: “The Ministry of Health has ordered a million Polio vaccinations”^11^). The remainder (13 %) of the items did not voice formal positions, but rather expressed concerns and complex positions of individual parents, or pro-vaccination and positive opinions (for example: “The Polio vaccination: Now everyone is hesitating”^12^).

Unlike the mass media which largely reflected the Ministry of Health’s formal positions, and rarely expressed alternative positions, the social media platform Facebook reflected the debates and discussions in the public, who used it to express their common positions and fears. As of the start of the Polio crisis in May 2013, Facebook exploded with criticism from angry parents [26]. Facebook was also filled with posts from many confused and hesitant parents, who did not know whose advice to follow [26]. Several anti-Polio vaccination groups were formed relying mainly on “ad hoc” risk communication such as information from popular sources that feature empirically uninformed claims about the extent, nature, and risks of vaccinations [19]. The primary mapping of the social media landscape revealed the following:

1. “Mothers Say No to the Attenuated Polio Vaccination”^13^ (4626 members)^14^
  This group was established on August 2, 2013 to oppose the “Two Drops” campaign. This campaign was described by this group as “unreasonable, irresponsible, and dangerous” and argued that Ministry of Health was silencing many cases of children who were vaccinated and then became paralyzed.
  The anti-vaccination activists who founded the group^15^ also claimed that the Ministry of Health’s approach lacked transparency and was deceiving the public, thus creating a “trust crisis”. The group declared its main aim to be “making hidden information available to the public”.
2. “Open Notepad – Parents for Transparency and Safety in Vaccinations”^16^ (4798 members)

This group was also set up in August 2013, soon after the “Mothers Say No to the Attenuated Polio Vaccination” group. Their official description states it was established “after a public battle over live Polio vaccine”. The group, which included parents and anti-vaccination activists, developed into one of the most active Facebook groups discussing vaccinations to this day. In August 2015 it changed its name to: “Vaccinations – an informed choice”. The group proclaims itself as being committed to informing the public that vaccinating children is a matter of personal choice, and that this choice should be based on informed judgment. Despite this statement, the group is actually an anti-vaccination group^17^ that urges parents to delay or reject vaccinations. Every vaccine is a matter of choice, according to this group, which is unequivocally against deferring to the government on vaccination decisions.

3. “Vaccines Inc.”^18^ (6200 members)

This longstanding anti-vaccination site (set up in 2010), declared itself to be “a reliable source of news and criticism about vaccinations and other health issues where the full truth is not being told”. The administrators of this page are practically all members of the previous group. This page was very active in the 2013 Polio crisis, and remains active to this day.

The posts by parents who turned to these anti-vaccination groups were thematically analyzed and classified into four main categories, and are listed here from the most popular to the least common:

1. Individualists: Some parents were furious to hear about the “sacrifice” they were requested to make; namely to expose their perfectly healthy children to risks, not for the sake of their own personal health, but for the sake of others, or society. The main perceived danger was the adverse effects of live attenuated vaccine (OPV). These parents resisted the notion that individuals need to protect society. They saw their children as individuals and were only concerned about them as individuals. As one angry mother wrote three days after the launching of the “Two drops” campaign: “ *Why should I, as a mother of a daughter who has been vaccinated with the IPV have to give her the OPV so that God forbid if my daughter is a carrier she won’t affect other children who have not been vaccinated… why do I need to take responsibility for others?”.* This mother rejected the moral argument of “for the greater good”, and was indifferent to the interpersonal and social harm that her daughter, if not vaccinated with OPV could cause others. This category is similar to what Navin [35] referred to as “hyper-individualism”. Another parent wrote: “*I just cannot understand what interest I could possibly have in vaccinating my son with the OPV if he has already been vaccinated with the IPV. Can someone explain to me why I should? Because right now I understand that in any case, even if he carries the virus in his stomach he is safe and will not actually get sick* ”.
2. Comments that expressed concerns about the safety of the OPV: these commentators were divided into three sub-categories:
  Those who adopted the typical stance of anti-vaccination movements worldwide, which see vaccines as ‘unnatural’ [36]. For example: *“I do not see vaccinations as innocent vitamins. There is much more to them and this is why I will never get vaccinated”*.
  Those who argued that the Ministry of Health was knowingly urging parents to give toxic substances to their children. A commentator wrote: “*Sounds nice and gentle and harmless… but the public has to inject/drink unnecessary substances which in some cases cause harm to their bodies…”.*
  Those who were very worried about the safety of the vaccinations and claimed that the vaccines may have contained hazardous ingredients which could pose a threat to their children’s health. For example, Eishton,^19^ an anonymous blogger, who conducted a thorough investigation including references from the UN and WHO, thus raised many questions regarding the vaccine.^20^
3. Comments which expressed distrust in the Ministry of Health: Some angry parents questioned the motives of the Israeli Ministry of Health, suggesting that the call for vaccination was meant to serve political interests rather than public health purposes. These parents did not consider the Ministry of Health as a medical authority as regards the necessity of the vaccination as a healthcare choice. These parents believed that children in Israel could do without the OPV, and that the Ministry of Health was forcing them to vaccinate their children to avoid pressure from the WHO. To be accountable to the WHO, the Ministry was needlessly requiring perfectly healthy children to get an unnecessary (in the best case) or a perilous (in the worst case) shot. As one parent wrote: “*I am not taking even the slightest risk where my children are concerned… They are not guinea pigs for the Ministry of Health*”.
4. Some argued that the Ministry of Health had purchased too much OPV. For example, a parent wrote:
*“I think that all the attempts to vaccinate our children are just an attempt on the part of the Ministry of Health to get rid of the surplus vaccines purchased when the virus was allegedly discovered in the south”*. Even parents who cannot be considered ‘vaccine denialists’; i.e. People who deny significant facets of the mainstream medical consensus regarding the risks and benefits of vaccines [35, 36], refused or hesitated to have their children vaccinated. This was mainly due to their distrust of the Ministry of Health [15]. As these parents saw it, the Ministry of Health was needlessly deterring them, while providing partial and contradictory information [15].
  The hesitating commentators also complained about the lack of consistency in the Ministry of Health’s messages, and its inability to provide clear and substantial information to the public. Many said this lack of consistency was what made them lose trust in governmental instructions as a whole. One parent wrote: “*Parents are confused […] no one gave an answer or presented the unambiguous implications of taking the oral vaccine*”.
5. Comments denying Polio as a viral disease: These parents dismissed the existence of Polio as an infectious disease. According to these parents, there is an interest-driven hype around the disease, whereas in fact this disease (1) does not exist (2) has long vanished from this world, and (3) is not caused by the Polio virus. For example, an active commentator on the “Vaccines Inc.” site wrote: *“My hope is to make you understand that […] there is no actual Polio disease, and no monster is chasing us”*.

## The Facebook group “Parents talk about the Polio vaccination”

According to the third writer, one of the founders of the group, “Parents talk about the Polio vaccination”^21^ was set up on August 14, 2013. All the types of comments and commentators mentioned above in the anti-vaccination groups were present in this group as well, but the administrators and their goals were very different.

This group constituted the most diverse public debate and discussion platform in the 2013 Polio crisis context, since it included participants from different professional and socio-economic backgrounds. Thirty-one percent of the commentators wrote only one item, but 12 % of the commentators were frequent contributors (i.e. people who contributed over 10 items). Of the frequent contributors, 55 % (*n* = 22) were physicians, who provided answers to the public’s questions.

Much like the other Facebook groups reviewed above, this group proclaimed itself neutral; in other words it did not have an official position concerning the Polio vaccination or any other vaccination. But while the other groups in fact disseminated an anti-vaccination ideology, this group identified itself as an evidenced-based pro- science group.

This Facebook group was set up by parents, physicians, and science enthusiasts. These parents stated that their sole concern was the wellbeing of their children, and wanted to present the facts and the best action plan for all parents in Israel. For the members of the group, the site served as their platform for debating, discussing, and question-presenting. As the group grew in numbers (as of November 12, 2013, it had 1741 members), it included physicians from the private sector (pediatricians and epidemiologists) as well as officials from the Ministry of Health, parents, pro-vaccination and anti-vaccination advocates, all arguing their case for or against the Oral Polio Vaccine.

**Fig. 1:**
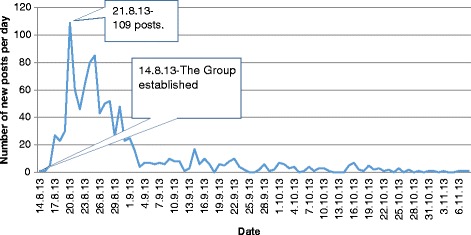
Number of new posts per day in the group “Parents talk about the Polio vaccination”

Figure 1 lists the number of posts in the group by date. The number of posts per day increased rapidly from the day the group was founded. This number peaked on August 21, with 109 daily posts (and their respective comments). From that day on, the number of posts per day slowly decreased until it reached a fixed level of a few (2-4) posts per day on November 12, 2013, the day we stopped collecting data. The data were collected continuously.

Most questions in the group were raised by concerned and skeptical parents, who primarily asked questions about their individual situation and personal issues. These questions were mainly answered by physicians and health administration officials, but also by other parents and sometimes even by anti-vaccination activists. According to the third author, who was one of the physicians who answered questions in the group (and also a co-founder and administrator of the group), they tackled misinformed opinions. They provided honest, reliable, and more importantly rapid information in a personalized manner.

### Who were the commentators?

Overall, 321 Facebook profiles were associated with the thousand-plus sampled items. The socio-demographic data for this sample provide insights into the commentators’ demographics. These were as follows^22^:

- Gender. Commentators’ gender was classified for 94 % of the sample: 36 % of the items for which author could be identified were written by men and 64 % by women.
- Education. Commentators’ level of education was identifiable for 34 % of the sample. Of these only 2 % had no academic education, 42 % held a bachelor’s degree, 8 % held a master’s degree, and 48 % held a Ph.D. or M.D. degree (both degrees were coded as one category).
- Occupation. Commentators’ occupation was retrievable for 37 % of the sample. Of these (independent of the previous paragraph) 13 % were physicians (*n* = 44), who were responsible for 909 of the items in the sample (909 out of 2289 items).
- Marital and parental status. Commentators’ marital status was identified for 30 % of the sample. Of these 98 % were married, and only 2 % were single, widowed, or single parents. It was possible to determine the parental status of 45 % of the sample. Of these 95 % had at least one child, and 5 % of the commentators did not have any children.

### General and personal queries and the ways these were answered by professionals and other participants

Parents in the group posted factual questions (e.g. ‘*what are the possible side effects of the vaccine?*’) and personal questions mentioning commentators’ relatives and loved ones, and their specific situations. “*I am pretty worried: yesterday at noon I left my two week old infant with the neighbor for a few minutes and went to get my older child who is 2.5 years old from kindergarten. When I came back, I was startled to discover that the neighbor accidently gave the baby the toddler’s pacifier (who had received the Polio vaccination). I called a medical center and it was closed for the holiday… Should I worry?*”. Our analysis shows that both general and personal queries were answered by professionals and other commentators. According to the third author, as well the other administrators of the group, each appeal received careful attention. If it was a personal appeal, one of the M.D.’s in the group (such as the third author of this paper) would provide an immediate and detailed personalized answer. “*Unequivocally there is no connection between thrombocytopenia (if indeed lack of thrombocytes is the problem in your case) and the Polio vaccination. Have you spoken to a hematologist at the hospital?*”. If it was a general appeal, many commentators would immediately start debates and discussions related to it. For example: “*Guys, the ‘Polio deniers’ were not convinced by thousands of documented paralysis cases*”.

The group provided space for dialogue between professionals and parents, many of which extended over long threads. It is unclear, however, to what extent those hesitating whether to vaccinate their children changed their minds as a result of these interactions. A telling example is a thread 98 comments long of a multi participant discussion with a worried mother, who concluded the discussion with the comment: “*I will probably take my child to get the vaccination today. [This was] after [I read] the document sent by X and after discussing the issue with my cousin who has read the research material thoroughly (she is familiar with research) and she spoke to physicians including alternative physicians, who recommended vaccination*”.

## Discussion

This paper described the social media component of the public debates and discussions during the 2013 Polio vaccination crisis in Israel. The findings show that while most of the web-based traditional media assessed in this report echoed the formal positions of the Ministry of Health, the social media (i.e. Facebook) served as a platform for lay audiences to express their opinions about the campaign’s merits or perils, and to get advice from peers, experts and pseudo-experts.

Vaccination-related messages on social media are an emerging issue, which is attracting growing attention in the scientific literature [4, 22]. In this context, the role of social media in disseminating anti-science and specifically anti-vaccination messages is very alarming and troubling [21, 22]. The 2013 Polio vaccination debates and discussions were used in this paper as a case-study to better understand how vaccination-related messages on social media can be described.

The 2013 Polio crisis can be better understood when set in its wider social context. The Health Ministry’s decision to launch a campaign to vaccinate children with OPV came at a time when there was little trust in governmental decisions in general in comparison to other Western democracies, or Israel in earlier eras [18]. According to the “Israeli Democracy Index 2014”, in 2013 only 37 % of Israeli Jews trusted the government, and only 28.4 % had faith in the mainstream media. It is important to note that the Israeli population is inherently divided into many ethnic and religious groups, which have different cultural assumptions [18]. The data showed that the Jewish population in general did not trust politicians, and felt that the government was not doing a good job in handling Israel’s problems [18]. It was not possible to obtain similar data about other specific ethnic and religious groups in Israel. These findings are consistent with a longstanding European trend of public skepticism toward political and social decisions [42]. Notwithstanding that European trend, the status of science and medicine as social institutions in Israel remains relatively sound [39, 45, 49]. However, groups such as “Parents talk about the Polio vaccination” are an important arena for the public to voice their stances, regardless of the degree of trust they have in science and medical institutions.

Trust (or the lack there of) in the Ministry of Health was only one of five reasons voiced by parents who turned to anti-vaccination groups. The other four, according to our findings, were individualism, concerns about th This highlights a problematic feature of the e safety of the OPV, the idea that the Ministry of Health had simply purchased too much of the OPV, and has to “get rid” of it, and finally, denying the existence of Polio. The targeting of OPV shows that some of the opposing parents in the case of the 2013 Polio crisis were not categorically against any vaccination, as is the case for classic vaccination opponents [36], but were made up in part of people who vaccinated their children, but opposed the OPV specifically.

The media ecosystem in Israel is heterogeneous, rich and diverse. There are a number of popular mainstream media platforms (such as Ynet, Mako, etc.), many sectorial media platforms (such as “Be-hadrey Haredim”, which is directed to the ultra-orthodox religious Jewish sector), and numerous social media platforms including Facebook, as well as a range of forums and interactive blogs [31]. Types of online and mobile media are expanding and traditional media are rapidly transforming their activity to online and mobile platforms. Thus the traditional media are still a solid component of the Israeli media landscape. Nevertheless, the Israeli media tend to direct considerable attention to certain subjects, for a relatively short period of time, and then cease to give them any attention [31]. The 2013 Polio crisis in the Israeli media was exposed for a short period of time, and its coverage was lowered to a minimum. The “Google trends” search clearly showed that the issue of Polio and Polio vaccinations was almost non-existent in the traditional online news media, except during our sampling period.

Now that the mainstream media are no longer the sole channel of communication between the government, the medical profession, and the public, social media enable discussions involving new authentic voices. These can be useful for informing policy-making [17, 42, 50]. In the case of “Parents talk about the Polio vaccination” these voices were more diverse than those appearing in the mainstream media, but were not representative of the entire population e.g. the participants were 64 % women and more educated than the average population. The relatively high level of education of the participants may be the result of the fact that most participants did not reveal their education level publicly (66 %). It is possible that commentators without a higher education simply provided less information about this aspect of their lives. Nevertheless, it has been found that attitudes toward vaccination are not correlated with education [47].

The value of revealing different public opinions and directly communicating with audiences is independent of their representativeness [27]. While analysing a health related public debate and discussion on social media is by no means a replacement for a representative survey it does provide an unobtrusive, updated and authentic view of the range of public attitudes and their trends.^23^ More importantly, social media health related public debates and discussions may provide a deliberative space for public and expert-public interactions.

Recent findings indicate that the Israeli public tend to put their trust in the government but choose not to comply with governmental instructions [48]. In our data, however, mistrust in the government was prevalent.

Social media have many advantages in comparison to mainstream media such as facilitating public participation in science and health communication [5, 28, 33, 50]. Also, as we have seen, social media hold the advantage of providing a platform for the public to debate, discuss, and voice their opinions and concerns. Nevertheless, social media clearly have negative aspects, most of which are true for traditional media too. One example is the uneven quality of information. The public, if exposed to misleading and biased information, will eventually develop trust problems and may choose not to follow formal medical advice [36].

Many questions remain unanswered. For example, is information or recommendations from accredited professionals treated differently by commentators than information or recommendations from lay people? Do groups such as ‘Parents talk about the Polio vaccination’, and social media in general, provide adequate tools to address vaccination opponents and conspiracy theories? How do the social media affect the decision to vaccinate? How can social media be harnessed to promote public health?

Further research is hence called for, to study what people actually learn in social media environments, what information and messages they remember and use for future reference, and the differences between those who are actively and passively engaged in social media debates and discussions.

## Conclusion

The social media serve as an outlet for the public, a platform for expressing doubts, worries, and criticism of political, medical and social issues. The social media thus constitute active and versatile debate and discussion-facilitating platforms in the context of vaccination discussions.

The importance of this paper lies in the presentation of the authentic voices of the public which should be acknowledged as useful for policy making purposes. While this article presented the findings of a descriptive study, its conclusions and recommendations should be further tested empirically.

The traditional online media supported the Ministry of Health in the Polio crisis of 2013. This in turn led to responses from both opponents and supporters in the social media. The social media were, in fact, the actual locus of debates and discussions (and the battle for public opinion). Our findings suggest that there are authentic voices which strongly object to formal health stances and recommendations. It has recently been shown that if decision makers wish to create an authoritative atmosphere they must convey their message by exhibiting professionalism, building trust and offering to share information [10].

Decision makers need to be cognizant of the authentic public voices we explored, and find ways to change public opinion in those forums where debates and discussions actually take place. Decision makers and formal authorities need to invest resources and manpower in answering questions and countering typical anti-vaccination messages when they are most likely to influence public decisions.

## Abbreviations

CDC, Centers for Disease Control; IPV, inactivated poliovirus vaccine; OPV, oral polio vaccine; WPV1, type 1 wild polio virus

## Footnotes

1 In a retrospective analysis of sewage samples, it emerged that WPV1 had been isolated as early as in February 2013 in a sample from Be’er Sheva [44].

2 Between 1957 and 1990, children in Israel were only vaccinated with OPV. IPV trials were administered in specific areas alone.

3 One out of 2.4 million children vaccinated with OPV (without IPV) may develop Poliomyelitis. This is not true of IPV (no risk) [12].

4 Social media such as Facebook and Twitter are internet applications that enable users to create and upload new content, comment on existing content, and share content with other users [4].

5 See: http://www.health.gov.il/NewsAndEvents/SpokemanMesseges/Pages/112012014_1.aspx.

6 According to a formal announcement from the Israeli Ministry of Health. See: http://www.health.gov.il/Subjects/vaccines/two_drops/Pages/Polio_updates.aspx.

7
http://newsroom.fb.com/company-info/

8 See: http://www.sciencedirect.com/science/journal/0264410X/30/25.

9
https://www.google.com/trends/explore#q=%D7%A4%D7%95%D7%9C%D7%99%D7%95&cmpt=q&tz=Etc%2FGMT-2.

10 If the post had more comments, which was true for about 200 of the 1039 posts making up the sampling frame, these comments did not appear in our database.

11 See: http://news.walla.co.il/item/2667355.

12 See: http://www.ynet.co.il/articles/0,7340,L-4415825,00.html.

13
https://www.facebook.com/groups/224882174329609/.

14 The number of members for all groups was recorded on October 21, 2015.

15 The group administrator’s husband petitioned the Supreme Court to halt the “Two Drops” campaign: http://www.health.gov.il/NewsAndEvents/SpokemanMesseges/Pages/29082013_1.aspx.

16
https://www.facebook.com/groups/1407224596178241/.

17
https://www.facebook.com/groups/1407224596178241/permalink/1664570533776978/

18
https://www.facebook.com/%D7%97%D7%99%D7%A1%D7%95%D7%A0%D7%99%D7%9D-%D7%91%D7%A2%D7%9E-111315228890879/.

19
https://www.facebook.com/Eishton.Blog/?fref=nf.

20
https://www.facebook.com/Eishton.Blog/posts/422956481143059.

21 See: https://www.‘Facebook’.com/groups/154989868031889/.

22 To collect the socio-demographic variables concerning the commentators, we accessed their personal Facebook profiles. We only coded variables for commentators with open profiles, where the data were publicly available. Thus the data were not always available.

23 There are many intelligence and commercial applications that monitor public attitudes online and on social media, see for example: https://blog.kiraradinsky.com/.
